# Takayasu's disease presenting as convulsive syncope which had been misinterpreted as epilepsy: a case report

**DOI:** 10.1186/1752-1947-4-352

**Published:** 2010-11-02

**Authors:** Bindu Menon, A Himabindu

**Affiliations:** 1 Department of Neurology, Narayana Medical College and Superspeciality Hospital, Chintareddypalem, Nellore-2 AP, India

## Abstract

**Introduction:**

Takayasu's arteritis is a chronic vasculitis mainly involving the aorta and its main branches. The disease has protean clinical manifestation ranging from asymptomatic to catastrophic illness.

**Case presentation:**

A 19-year-old woman of Asian origin was referred to our neurology out-patient department for the management of refractory seizures. She reported several episodes of a loss of consciousness with tonic posturing when she assumed an upright position, which was accompanied by constitutional symptoms. A clinical examination showed orthostatic hypotension and an investigation confirmed the diagnosis of Takayasu's disease with presentation as convulsive syncope.

**Conclusion:**

Our case highlights the importance of a thorough clinical history and physical examination in order to distinguish events mimicking epileptic seizure. We also describe an unusual presentation of Takayasu's disease with convulsive syncope and systemic constitutional symptoms.

## Introduction

Takayasu's arteritis (TA) is an inflammatory and stenotic disease of medium-sized and large-sized arteries characterized by a strong predilection for the aortic arch and its branches. Symptoms of the disease start with non-specific constitutional symptoms in the first stage to organ specific ischemic symptoms in the second stage. Occasionally there is no clear demarcation between stages, and diagnosis can be difficult in such a setting. We report the case of a 19-year-old woman who was referred to our neurology out-patient department for recurrent episodes of loss of consciousness and systemic constitutional symptoms.

## Case presentation

A 19-year-old Asian woman was referred to the department of neurology for the management of recurrent seizures. She had been having recurrent episodes of a loss of consciousness. On detailed enquiry she revealed that these episodes were present immediately on changing to an upright posture from a lying position. She would lose consciousness, become pale, and fall to the ground. She would then experience tonic posturing of the limbs lasting for a few seconds. She would immediately regain consciousness in the supine position, with no postictal drowsiness. There were several similar episodes a day, and this had persisted for the last three months. Her symptoms persisted even after treatment with phenytoin, phenobarbitone, and sodium valproate, with no decrease in the frequency of episodes. She also complained of fatigue, malaise, myalgia, giddiness, fever, decreased appetite and weight loss over the preceding three months. Her medical history was not significant. There was no previous or family history of tuberculosis.

Physical examination revealed a thin built, frail and anxious patient. She was afebrile. Her radial pulses were very feeble while lower limb pulses felt normal. Her bilateral carotid pulses were also feeble. Upper limb blood pressure (BP) was measured (with difficulty) as being 72/58 mmHg in both upper limbs in a supine position. Standing BP was 50/48 mmHg in the upper limbs, suggestive of orthostatic hypotension. Lower limb blood pressures were130/70 mmHg bilaterally. The results of cardiac examination were normal. Her higher mental functions and fundi were normal. There was no cranial nerve, motor or sensory deficit.

**Figure 1:**
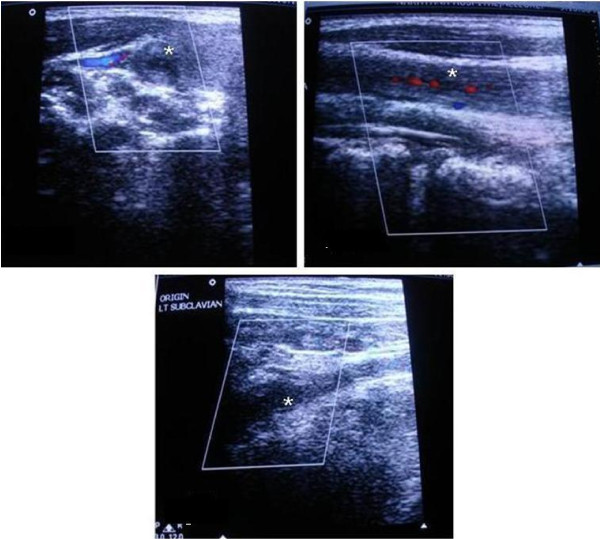
Color Doppler images of right (A) and left (B) common carotid arteries (*) show diffuse wall thickening and a streaky flow in the lumen. The origin of left subclavian artery (C) shows no flow.

**Figure 2:**
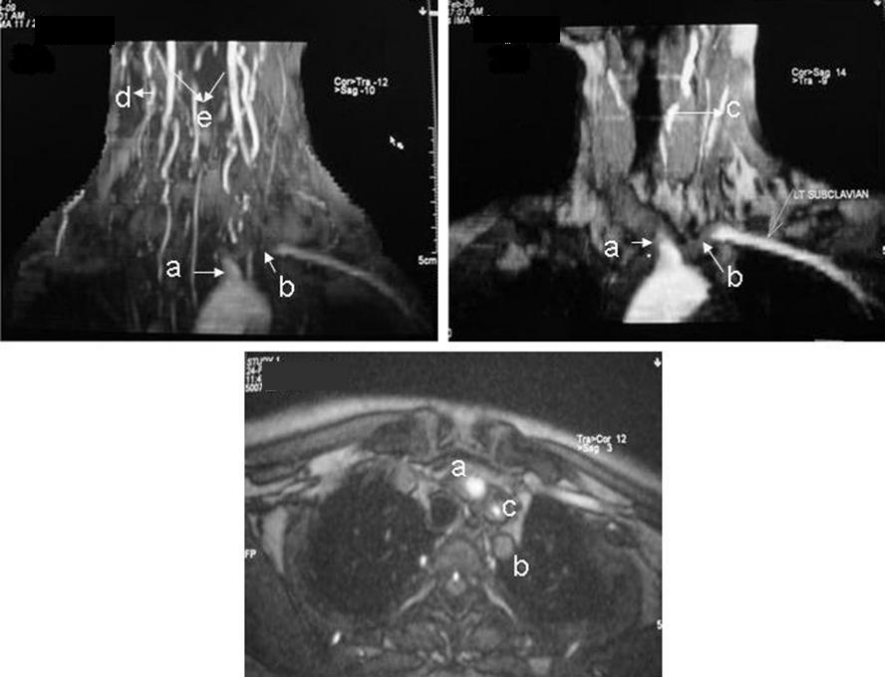
**Maximum intensity projection (A) and thin coronal reconstruction images (B) from two-dimensional 'time of flight' magnetic resonance angiography show no flow in the brachiocephalic artery (a) beyond the origin**. The origin of left subclavian artery (b) does not show a flow signal. The left common carotid artery (c) shows a streaky flow. The image in (A) shows multiple collateral vessels (d) along with normal flow in bilateral vertebral arteries (e). Axial reconstruction image (C) shows diffuse wall thickening of arteries with luminal narrowing.

Blood test results indicated anemia with hemoglobin of 9 gm/dlL, an elevated erythrocyte sedimentation rate (ESR) of 42 mm/hour and a total leukocyte count of 13,400 cells/mm^3^. Her C-reactive protein level was elevated at 26 mg/L. Tests for plasma sugars, liver function tests, renal function tests, HIV/hepatitis B virus surface antigen (HBsAg) antibodies, hepatitis C virus antibodies, and Venereal Disease Research Laboratory test results (protoplasmic-staining anti-neutrophil cytoplasmic antibodies (p-ANCA), cytoplasmic staining ANCA (c-ANCA) and Hbs Ag) were all negative. Her chest X-ray, electrocardiography, echocardiography and electroencephalography findings were normal. A color Doppler of the carotid vessels showed right (Figure 1A) and left (Figure 1B) common carotid arteries showing diffuse wall thickening and streaky flow in the lumen. The left subclavian origin showed no flow (Figure 1C). Bilateral brachial, radial and ulnar arteries showed low velocity monophasic flow. Renal Doppler results showed normal renal vessels. The abdominal aorta was not imaged. A magnetic resonance angiography (MRA) study of the carotid and vertebral vessels was performed with a 0.35 Tesla Siemens Magnetom C device (Siemens, Mumbai, India). Maximum intensity projection (Figure 2A) and coronal reconstruction images (Figure 2B) from two-dimensional 'time of flight' MRA showed no flow in the brachiocephalic artery beyond the origin. Origin of left subclavian artery did not show a flow signal. The left common carotid artery showed streaky flow. Multiple collateral vessels were seen. The vertebral arteries were normally visualized.

An axial reconstruction image (Figure 2C) showed diffuse wall thickening of arteries with luminal narrowing.

Our patient was diagnosed as having TA, fulfilling four of the six criteria for diagnosis: development of symptoms or findings related to TA at age ≤40 years, decreased pulsation of one or both brachial arteries, arteriographic narrowing or occlusion of the primary branches of the aorta, and a difference of >10 mmHg in systolic blood pressure between arms [1]. The results of laboratory investigations for anemia, elevated ESR and C-reactive protein were suggestive of active disease. She was hospitalized and prescribed complete bed rest. Prednisolone (40 mg/day) along with antacids were given. Our patient started feeling better after a week and was therefore gradually mobilized. She did have episodes of giddiness, but there was no loss of consciousness or tonic posturing of limbs. She was discharged after two weeks. After a year, at follow-up she had made a clinical improvement with no constitutional symptoms or syncopal seizures but her radial pulses remained feeble. There was no evidence of postural hypotension.

## Discussion

TA is a chronic vasculitis that mainly involves the aorta and its main branches (the brachiocephalic, carotid, subclavian, vertebral and renal arteries), as well as the coronary and pulmonary arteries. The disease is most commonly seen in Asian and Latin American countries [2]. The incidence of TA is about 2 in 10,000 person-years [3]. The female/male ratio varies from 9:1 in reports from Japan to 1.3:1 in India [4]. The underlying pathology is inflammation leading to stenosis, blockage or aneurysm formation. The disease is also called the pulseless disease because of the difficulty in detecting the peripheral pulses.

The etiology of the disease is still poorly understood. The clinical presentation is widely heterogeneous depending on the involvement of the vessels. The disease has three stages, in which there is an initial pre-pulseless stage predominated by systemic constitutional symptoms. As a result of the non-specificity of the symptoms the diagnosis is often delayed until the next stage of vascular insufficiency. The second stage may be devoid of any signs of inflammation. Hypertension due to renal artery stenosis, retinopathy, aortic regurgitation, aneurismal enlargement of aorta, congestive cardiac failure, postural dizziness, amaurosis, transient ischemic attacks and stroke are some of the presenting features in the second stage. The third stage is the stage of quiescence. Collateral circulation develops because of the chronic nature of the illness. Neurological manifestation is seen in about 20% of cases. The initial manifestations are predominantly ischemic in nature because of the stenosis of the vessels. Seizures as an initial manifestation have been reported but are rare [5]. Syncopal seizures have not been reported. A MeSH database searches with the headings 'Syncope', 'convulsion' and 'Takayasu's arteritis' did produce any reported studies.

Our patient had stenosis of the major branches of the aorta, brachiocephalic, subclavian and left common carotid artery. The cerebral perfusion was being maintained by the collateral circulation. The differential diagnosis of episodic neurological dysfunction included a vast range of disorders. The loss of consciousness, with tonic posturing, led to the misdiagnosis of epilepsy. However, these symptoms were suggestive of convulsive syncope because: all episodes had a postural component, episodes were only for few seconds and there was no postictal drowsiness, our patient immediately regained consciousness in the recumbent position and documented orthostatic hypotension was found on examination. Syncope is transient loss of consciousness due to cerebral hypoxia. This may be accompanied with convulsive episodes. Convulsive syncope merely represents a variant of syncope, which is accompanied by tonic or myoclonic activity.

The differential diagnoses of TA include tuberculosis, temporal arteritis, atherosclerosis, fibro muscular dysplasia and syphilitic aortitis. Apart from syphilitic aortitis, which affects the aorta with calcification, all the other differentials have a predilection for other vessels. The gold standard of investigation was considered to be angiography, but non-invasive Doppler and MRA can provide equally good results [1].

In the active stage of the disease, judged by systemic symptoms, glucocorticoids are the mainstay of treatment. This is thought to halt the inflammation and further stenosis in vessels. Serological tests have not so far proved helpful in differentiating the active from the inactive stage [6]. Surgery is indicated in certain patients with inactive disease.

## Conclusion

Misdiagnosis of epilepsy remains a major clinical problem. We presented this case in order to highlight two findings: the importance of a detailed clinical history and examination, which will help to determine whether or not an epileptic seizure actually occurred in a patient and to differentiate seizure mimics and the absence of stage-wise progression of the symptoms in TA which can create a diagnostic dilemma.

## Consent

Written informed consent was obtained from the patient for publication of this case report and accompanying images. A copy of the written consent is available for review by the Editor-in-Chief of this journal.

## Competing interests

The authors declare that they have no competing interests.

## Authors' contributions

BM diagnosed the case of TA, collected the requisite literature and analyzed and interpreted the data from our patient. AB performed and interpreted the radiological examination. Both authors read and approved the final manuscript.
